# Current News

**Published:** 1889-05

**Authors:** 


### Current News.

Attend the dental conventions this year.

Read "Current News" in this and last issue for notices of summer meetings.

Write to Dr. Crouse, and he will make an appointment to meet you at some of them, and "take" you into the *Dental Protective Association.*

Don't wait until an injunction has been served upon you. It will then be too late, and you will have to settle or fight alone.

Don't forget that the Dental Protective Association will bear all the expense of suits brought against individuals after they have become members of the association.

Send ten dollars to Dr. J. N. Crouse, 2231 Prairie Avenue, Chicago, and dismiss all care. He will your burden assume, and make the pathway of the agent of the International Tooth-Crown Company bristle with thorns.

The State of Minnesota has just passed a most excellent law governing the practice of dentistry within her borders. The new law takes the place of the one passed in 1885, and is considered to be a decided improvement.

Dr. A. H. Thompson gives it as his experience that bichloride of mercury turns a tooth as black as his hat! Has any one else had similar effects? No one was found at the Chicago meeting to corroborate it.

Dr. Sudduth says that in opthalmology silico-fluoride of soda is replacing boric acid, after much careful experimentation. It is non-irritant, non-poisonous and is a good disinfectant.

Dr. A. W. Harlan is of the opinion, though empirically, that the essential oils possess properties hitherto unsuspected or overlooked, notwithstanding their recognized value as household remdies.

Dr. Black says the whole subject of antisepsis is young yet. As we proceed in our investigations and grow in knowledge, we are liable to have to take out whole blocks from our foundation, and build anew; flaws may be found in our technique which will prove our results all wrong; but we must continue to work at it.

Dr. J. H. Wooley says that after the work of disinfection has apparently been done by drugs, and we consider the root in condition to fill, application of a heated broach will frequently reveal the characteristic odor of sulphurated hydrogen showing that decomposition still lurks in the canal. This can be more effectually overcome by the aid of the heated broach than by any other method.

Dr. Curtis says that women accustomed to the quiet of home frequently associate noise with pain, and that our operations for them should be as quiet as possible; to many the filing and cutting in the laboratory are not to be endured. So of smell; the odor of creosote recalls a howling toothache; that of chloroform, the lancing of a felon; of iodoform, the dressing of a painful wound. Let nothing about the office recall these painful associations.

Dr. C. M. Bailey says that while the experiments of Dr. Black prove positively that iodoform neither destroys nor inhibits the growth of microbes, even in the undisolved powder; yet we find in clinical practice that it *does* prevent the formation of pus! Iodoform in eucalyptus oil—both valueless according to Dr. Black's tables—we find will most perfectly sterilize an abcessed root and induce perfect healing. It may be empirical but it succeeds!

Dr. T. E. Weeks, Minneapolis, holds that after all *mind cure* in one form and another is the great dependance in operating on sensitive dentine. Gain the confidence of your patients, show them that you know just what to do, and just how to do it. To be able to do this you must have a thorough knowledge of tooth structure, of both its organic and its inorganic domains, so that you will run no risk of passing the boundary line; otherwise it is like playing with fire.

Dr. Pruyn warns earnestly against the fascinating and seductive dangers of cocaine. He says the cocaine habit is far worse than alcohol or morphine; the demand will not be denied. Especially he warns against the danger of self-injection.

Dr. Pruyn says that no man should use cocaine in his dental practice who has not studied its effects upon the lower animals, even to death. In that way only can he recognize all the symptoms and know their value and indications.

Dr. Martindale says that in necrosis of the lower jaw, the external plate is frequently bulged out by the inflammatory effusion between the plates. In some cases the effusions force an outlet and the plate collapses; in others the effusion organizes new products and the cavity becomes filled with new bone, and a permanent bony tumor is the result.

In obtunding sensitive dentine by electrolysis, Dr. McGraw uses a 12 per cent. solution of cocaine in absolute alcohol and alum. This is placed in the cavity on a pledget of cotton, the positive pole being placed on the cotton and the negative on the cheek; the galvanic current from four cells is then passed through for three minutes, and repeated after an interval of three minutes. He gets the anesthetic action of the cocaine and the dessicating effect of the alcohol.

Dr. L. L. Davis, Chicago, says that so far, the porcelain fillings that he has seen, which were made by Dr. Land's method, with platinum back, are not what he would want either for himself or for his patients.

Dr. D. F. McGraw enumerates among the advantages offered in obtunding sensitive dentine by electrolysis, that it is not patented, that it is safe with no possible evil results, that it necessitates no high priced drugs.

Dr. G. E. Weeks, Minneapolis, in his paper on sensitiveness of dentine and its control, read at the Chicago Dental Society meeting, said that nominally the pulps can only transmit a sense of pain from change of temperature but that the degree of temperature can only be distinguished by the aid of the tongue and lips. It has no tactile sense.

Dr. Comstock holds that the artistic adaptation in prosthetic dentistry can be systematized and taught as it is based entirely on well-known art principles and a knowledge of facial anatomy, and the science of physiognomy or facial expression.

Dr. C. Thomas, Des Moines, Iowa, makes his own porcelain inlays and fillings, for all kinds of cavities, contouring corners of incisors, building up molars, etc. No matter what the shape of the cavity he burnishes into it platinum ribbon, forming a matrix in which bakes the filling. A platinum pin, or two if necessary, is baked in the *patch* a corresponding pit being drilled in the tooth for the reception of the pin. The fillings are set with oxy-phosphate cement.

Dr. Allport regards the attainment of the ideal in prosthetic dentistry to depend upon its taking rank as an art, with painting and sculpture. It must be recognized as the special province of only the very few who can hope to attain the requisite perfection in artistic productions. Our colleges should understand this and teach it as such an art, not to be relegated to an obscure mechanical workman back in a labratory workshop.

Dr. Dorance, Michigan, thinks that from the superior density and better color of artificial teeth, better results will be obtained from fillings made from pieces of broken teeth than from inlays made from the model of the cavity and baked in a platinum matrix, though far more skill is required to grind and adjust the former. He does not think the method of universal application at present, but considers it well worthy the thought and efforts of young practitioners, as *the* filling of the future.

In the discussion of Dr. Andrews' lantern exhibit, Chicago, Dr. Black said that while there could be but one opinion as to the function of microbes, his own work made him less positive on the subject of calco-globulin. He had observed the thin onion-like spherical formations in mal-formed teeth where all is chaos, in softened bone after inflammation of the pulp, about the ends of the long bones in young animals; we might expect some abnormality in those positions, or when left to chemical action in jars in the laboratory where life forces have no play. But as shown in the illustrations shown by Dr. Andrews he thought it might possibly be due to some post-mortem change. In his own work he had found that in the use of certain reagents he got that formation very frequently, while in other modes of work he got none.

When, from excessive brushing or other causes, the gums have receded leaving the necks of the anterior superior teeth exposed, Dr. A. H. Thompson (Topeka), makes an almost imperceptible restoration to natural appearance by inlays of gum-colored porcelain, to which he gives the fullness of the natural gum. By careful selection of the proper côlor, making from the gum-portion of artificial teeth, grinding to the proper thinness from the tooth-body side, and setting in cement so colored as to still further assist the disguise, a most perfect restoration to normal appearance is made, as was demonstrated in the clinic given at the Chicago Dental Society meeting.

---

The Thirteenth Annual Meeting of the New Hampshire Dental Society will be held at Concord, June 18 and 19, 1889. All members of the profession invited to attend. Reduced railroad fares. The Board of Censors will meet any candidate for license Monday evening, June 17th, at the office of the Secretary.

EDWARD B. DAVIS, *Secretary.*

---

CHICAGO DENTAL SOCIETY.

